# Performing Chest X-Rays at Inspiration in Uncooperative Children: The Effect of Exercises with a Training Program for Radiology Technicians

**DOI:** 10.1155/2014/312846

**Published:** 2014-07-09

**Authors:** Heinz-Jakob Langen, Christiane Kohlhauser-Vollmuth, Corinna Sengenberger, Johann Bielmeier, Renate Jocher, Martina Eschmann

**Affiliations:** ^1^Department of Radiology, Medical Mission Hospital, Salvatorstr. 7, 97067 Wuerzburg, Germany; ^2^Department for Paediatric and Adolescent Medicine, Medical Mission Hospital, Salvatorstr. 7, 97067 Wuerzburg, Germany; ^3^Department of Nucelarmedicine, Marienhospital Stuttgart, Böheimstr. 37, 70199 Stuttgart, Germany

## Abstract

*Objective*. It is difficult to acquire a chest X-ray of a crying infant at maximum inspiration. A computer program was developed for technician training. *Method*. Video clips of 3 babies were used and the moment of deepest inspiration was determined in the single-frame view. 12 technicians simulated chest radiographs at normal video speed by pushing a button. The computer program stopped the video and calculated the period of time to the optimal instant for a chest X-ray. Demonstration software can be tested at website online. Every technician simulated 10 chest X-rays for each of the 3 video clips. The technicians then spent 40 minutes practicing performing chest X-rays at optimal inspiration. The test was repeated after 5, 20, and 40 minutes of practice. *Results*. 6 participants showed a significant improvement after exercises (collective 1). Deviation from the optimal instant for taking an X-ray at inspiration decreased from 0.39 to 0.22 s after 40 min of practice. 6 technicians showed no significant improvement (collective 2). Deviation decreased from a low starting value of 0.25 s to 0.21 s. *Conclusion*. The tested computer program improves the ability of radiology technicians to take a chest X-ray at optimal inspiration in a crying child.

## 1. Introduction

In pediatric radiology especially, quality control such as the improvement of chest X-ray examinations is very important [1]. In addition to the technical parameters of quality, the timing for taking an X-ray during breathing is very important for diagnostic evaluation. The main focus in conventional chest X-ray examinations in children is the exclusion or proof of pneumonic infiltration. In particular, chest X-ray examinations in babies and young children demonstrate characteristic changes like infiltration only at deep inspiration [2]. Babies and small children are not able to follow breathing commands. A good chest X-ray examination depends among other things on the ability of the technician to take the X-ray of a potentially crying child at an optimal instant (deep inspiration). The moment for taking an X-ray is very short. The respiratory frequency in an adult is 16 breaths per minute and increases to 80 breaths per minute in an ill infant. That means that the diaphragm moves up and down in less than a second [3]. The goal of this study was to evaluate a training program using a self-developed video game practicing the taking of a chest X-ray at the optimal moment. The improvement of the reaction time using the training program was demonstrated in an earlier study [4]. This study examined how much training time is needed to improve reaction time and whether the improvement is shown for all children (i.e., crying and sleeping children) and in all test subjects.

## 2. Materials and Method

### 2.1. Patients

The study involved a training program and a test program containing video clips of different children. For the study videos were taken with 24 images per second of six children with consent from the parents. The videos of the patients were all obtained in supine position on the examination table or in bed. While filming a crying and a sleeping newborn was no problem, it was not possible to find a sporadically crying newborn. Therefore videos were taken of an older child for that breathing type. In detail, video clips were taken of a regularly crying (meaning that the duration of a respiratory cycle during the investigated time remains the same) 25-day-old newborn (training child 1), a sporadically crying (meaning that the duration of a respiratory cycle during the investigated time does not remain the same) 17-month-old child (training child 2), and a sleeping 8-day-old newborn (training child 3). Additionally, to evaluate the training outcome, a test program was created. Therefore videos were taken of a regularly crying 6-month-old infant (test child 1), a sleeping 5-month-old infant (test child 2), and a sporadically crying 15-month-old child (test child 3).

### 2.2. Computer Program

**Figure 1:**
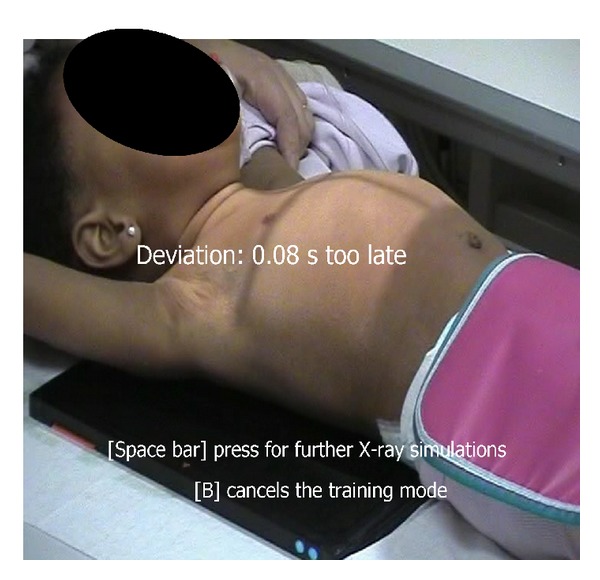
Frozen frame after simulation of a chest X-ray in a crying infant (17-month-old child) (training child 2) after the space bar is pressed. The center of the image shows that the running video was stopped 0.08 s too late for an X-ray at deepest inspiration.

With a video editing program (MAGIX Video Deluxe 2006 plus) in video clips with a duration of 36 (training child 1), 22 (training child 2), and 11 (training child 3) seconds, and 47 (test child 1), 42 (test child 2), and 47 (test child 3) seconds, the moment of the deepest inspiration was visually determined using the single-frame view. A computer program allowed stopping of the normal running video with the push of a button connected to the computer by a USB plug. The video stopped with a delay of 0.006 s corresponding to the standard technical delay when taking an X-ray from the prepare mode. The delay in the computer program can be changed according to the different X-ray devices. After the video was stopped, the computer program calculated the period of time from the stopping of the video to the moment of a chest X-ray of optimal inspiration. When the video was stopped too early, the period of time to the optimal moment got a negative sign. If the video was stopped too late, the sign was positive. In the background the period of time was calculated with a precision of nearly 0.04 seconds approximately corresponding to the duration of one picture at 24 pictures per second (Figure 1). The computer program was created using the computer language Delphi (Embarcadero Technologies; San Francisco; Version Delphi 2007 for Win32. A free video player (mplayer; Download: http://www.mplayerhq.hu; Version: 1.Opre8-3.4.2) was also used. Demonstration software of the “training program for radiology technicians” can be tested at the website http://www.tprt.de/.

### 2.3. Test Setting

Test subjects included twelve pupils from the school for radiology technicians in the second and third year of training without experience in pediatric radiology. Test subjects number 5 and number 12 were male. All others were female. First a baseline test (test 1) was performed with all 12 test subjects. Therefore, each test subject simulated 10 chest X-rays for each of the 3 test video clips. If a video clip ended before completion of the 10 simulations, the video was started again. In the background the deviation from the optimal moment for an X-ray at inspiration was recorded. Afterwards the test subjects used the training videos to practice taking X-rays at optimal inspiration in a manner similar to playing a computer game. During the exercise the period of time to the optimal instant was shown to the test subjects after every simulated X-ray. The test was repeated after 5 minutes (test 2) and additional 15 minutes (test 3). 2 to 5 days after the first exercise day, the test subjects trained again for 20 minutes before testing (test 4). Following a training break of one week, the final test (test 5) was conducted without prior training to demonstrate the long-term effects of the training program. Changes were evaluated using the *t*-test for independent samples and the Dunnett test [5]. A *P* value < 0.05 was considered to be significant. The period of time to the optimal instant is recorded as mean of the absolute values without signs ± standard deviation.

## 3. Results

Initial evaluations showed a significant interindividual difference in the results of the test subjects. Some of the test subjects demonstrated an important improvement due to the training, while others scored well from the beginning but showed only minimal improvement. To demonstrate the different effects of the training program in both groups, the test subjects were divided in 2 groups. 6 test subjects, who showed significant improvement in the regularly crying child based on the *t*-test for independent samples, were summarized in collective 1. The other 6 test subjects without significant improvement were summarized in collective 2.

**Figure 2:**
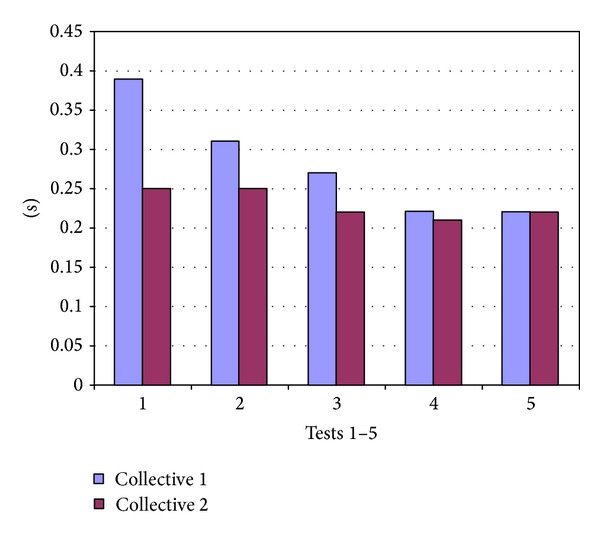
Deviation from the optimal inspiration moment in seconds in test 1 before training and in tests 2 to 5 after training in collective 1 and 2.

In collective 1 the test subjects missed the moment for an X-ray at maximal inspiration by 0.39 (±0.35) s in test 1 while the deviation was significantly reduced to 0.22 (±0.26) s after training in test 4 (*P* < 0.001). In collective 2 the deviation changed from 0.25 (±0.26) s in test 1 to 0.21 (±0.14) s after training in test 4. The changes were not significant (Dunnett test *P* = 0.125) (Figure 2).

**Figure 3:**
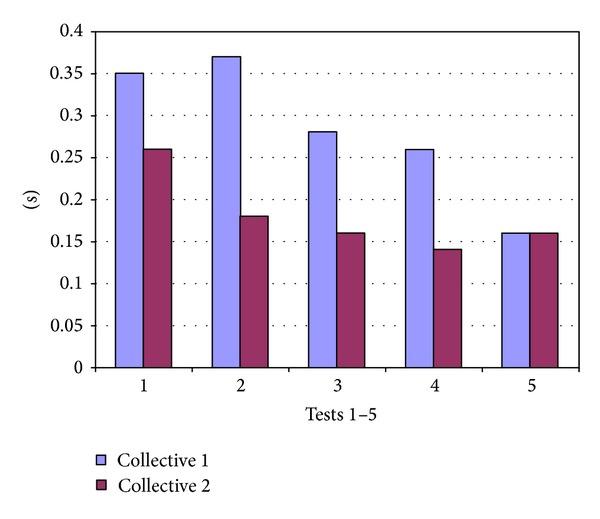
Standard deviation in seconds in test 1 before training and in tests 2 to 5 after training in collective 1 and 2.

The greatest improvement in accuracy was demonstrated after only 5 minutes of training and increased after further training compared to test 1. In collective 1 the deviation from the optimal instant decreased from 0.39 (±0.35) s in test 1 to 0.31 (±0.37) s in test 2 (*P* = 0.04) to 0.27 (±0.28) s in test 3 (*P* = 0.001) and to 0.22 (±0.26) s in test 4 (*P* < 0.001). In collective 2 the changes were not significant at the high starting point. The mean deviation from the optimal instant was 0.25 (±0.26) s in test 1, 0.25 (±0.18) s in test 2, 0.22 (±0.15) s in test 3, and 0.21 (±0.13) s in test 4 (Figure 2). The mean standard deviation decreased as a result of training in both collectives (Figure 3).

**Figure 4:**
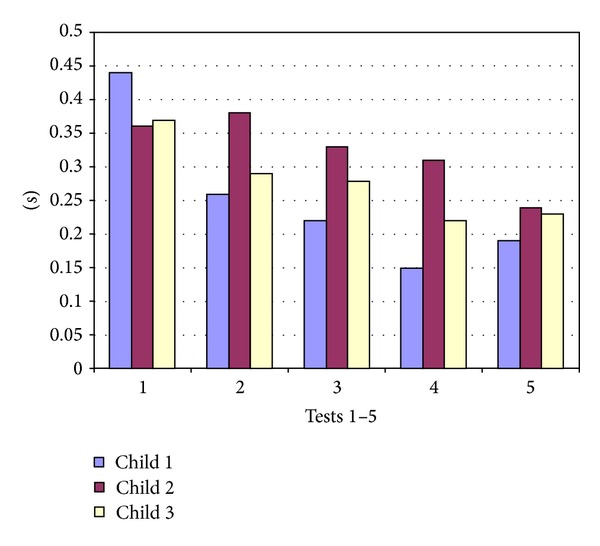
Deviation from the optimal inspiration moment in seconds for child 1 (regularly crying), child 2 (sleeping), and child 3 (sporadically crying) in test 1 before training and in tests 2 to 5 after training in collective 1.

The greatest improvement in accuracy was shown in the regularly crying infant (test child 1). In collective 1 the deviation from the optimal instant decreased on average from 0.44 (±0.34) s in test 1 to 0.15 (±0.14) s in test 4 (*P* < 0.001). The most significant changes were demonstrated after only 5 minutes of training with a mean deviation from the optimal instant of 0.44 (±0.34) s in test 1 to 0.26 (±0.20) s in test 2 (*P* < 0.001). Also significant was the improvement from 0.22 (±0.17) s in test 3 to 0.15 (±0.14) s in test 4 (*P* = 0.008). Test child 2, the sleeping child, resulted in the least improvement in the mean deviation from the optimal instant from 0.36 (±0.41) s in test 1 to 0.31 (±0.39) s in test 4. The changes however were not significant in this child. Test child 3, the sporadically crying infant, also yielded insignificant improvement in the mean deviation from the optimal instant from 0.37 (±0.28) s in test 1 to 0.22 (±0.16) s in test 4. The greatest changes were seen from test 1 0.37 (±0.28) s to test 2 0.29 (±0.27) s and from test 3 0.28 (±0.20) s to test 4 0.22 (±0.16) s. However, the changes were not significant (Figure 4).

**Figure 5:**
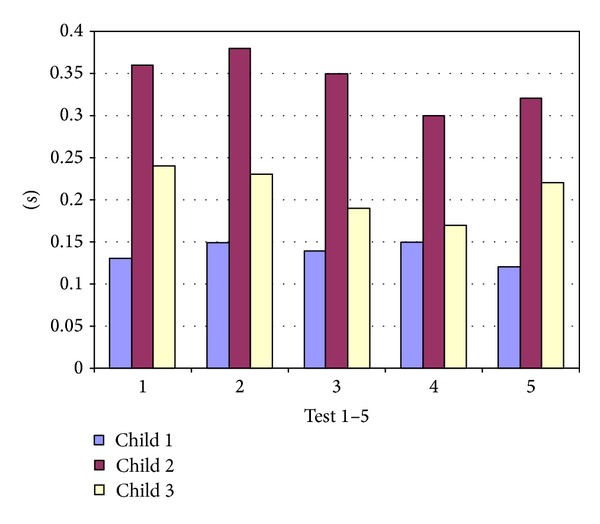
Deviation from the optimal inspiration moment in seconds for child 1 (regularly crying), child 2 (sleeping), and child 3 (sporadically crying) in test 1 before training and in tests 2 to 5 after training in collective 2.

In collective 2 the smallest deviation from the optimal instant for an X-ray was seen in the regularly crying infant (test child 1). The mean deviation changed from a lower starting point of 0.13 (±0.1) s in test 1, not significant, to 0.15 (±0.11) s in test 4. In test child 2 (sleeping child), the mean deviation improved from 0.36 (±0.35) s in test 1 to 0.30 (±0.16) s in test 4. These changes were also not significant. In test child 3 (sporadically crying infant), the mean deviation improved significantly (*P* = 0.021) from 0.24 (±0.21) s in test 1 to 0.17 (±0.09) s in test 4 (Figure 5).

The training program also yielded long-term effects as evidenced by the results from test 5 which were performed after a training break of one week. In collective 1 the mean deviation from the optimal instant for an X-ray for all three children of 0.22 (±0.26) s in test 4 and 0.22 (±0.16) s in test 5 showed no significant change. In collective 2 the change of the mean value from 0.21 (±0.14) s in test 4 to 0.22 (±0.16) s in test 5 was also not significant (Figure 2).

## 4. Discussion

Conventional chest X-rays are commonly used in children, especially in the neonatal and pediatric intensive care units. Since conventional X-ray diagnosis provides valuable information with minimal radiation exposure and relatively low costs, it is the foundation for further imaging methods such as fluoroscopy, MRI, or CT [6, 7]. Deep inspiration results in a better chest X-ray, with a better evaluation of the lung parenchyma [2]. Inadequate inspiration leads to condensation of the lung structures, which can simulate pathological findings [8]. While adults take a deep breath on demand and stop breathing when the chest X-ray is taken, this is not the case in small children and babies. Nearly half of the children requiring a chest X-ray are of an uncooperative age [6]. Radiology technicians have to estimate when the child will reach the moment of maximal inspiration during the respiration cycle. The diaphragm passes through the whole respiration cycle in 1.5 seconds or less if the respiration frequency reaches 60 breaths per minute or higher. In a crying child a diaphragm speed up to 100 mm/s is possible [6]. The radiology technician has a very limited opportunity to correctly estimate the optimal moment for a chest X-ray [2]. Despite the high importance of good chest X-rays in babies and small children and the difficulties regarding taking a chest X-ray at the moment of deepest inspiration, we only know of one study addressing improvement of this issue. Claesson and Olsson [9] used transthoracic impedance as a triggering device for chest X-rays. Although they reported good results, this technique was not developed further. Experienced radiology technicians advise trainees to breath with the child in order to determine the point of deepest inspiration for the chest X-ray more exactly. The newly developed program makes a positive contribution, especially for inexperienced radiology technicians and trainees. They can optimize their technique without radiation exposure.

On average all test subjects improved their accuracy during the tests as a result of training. However, the changes were significant only in collective 1 (subjects who showed significant improvement in the regularly crying child). This may be due to the fact that the test subjects in collective 2 (subjects without significant improvement in the regularly crying child) began with better performance and therefore only minimal improvement was possible. Probably the test subjects in collective 2 had such a good reaction and learning capacity that they understood after the first introduction how to determine the optimal instant for a chest X-ray at inspiration. However, the training program was also useful in this subgroup because the accuracy improved even if not significantly. The program provided the greatest benefit to the test subjects of collective 1. The initial accuracy achieved by this subgroup improved significantly due to training.

After training the smallest deviation from the optimal moment to take a chest X-ray was achieved in the regularly crying child. The test subjects were probably better able to adjust to regular breathing and to take the optimal moment more reliable than to the quick breathing of the sleeping infant. The results correlate well with an earlier study [4]. In our study the worse results were seen in the sleeping child followed by the sporadically crying child. It is conceivable that X-rays can be taken more exactly at inspiration in this subgroup if the child is made to cry at regular intervals by uncomfortable stimulus. However, the moment of deepest inspiration is also determined more exactly after training in a sporadically crying child, but the results were clearly worse compared to the regularly crying children. In most of the cases a decrease in the standard deviation was shown after training. This means that the test subjects achieved the optimal instant for a chest X-ray with higher accuracy and the number of outliers decreased.

The greatest improvement in the deviation from the optimal instant to take a chest X-ray was seen after only 5 minutes of training. However, even after 40 min of training, smaller—sometimes significant—improvements can be demonstrated. The training time required for the test subjects to reach their optimal performance varies greatly. A fixed training time cannot be recommended. Every test subject needs to practice until maximum ability to take a chest X-ray at deepest inspiration has been reached. The good news is that these skills are retained for a substantial period of time.

## 5. Conclusion

In summary the tested training program helps radiology technicians improve their ability to take a chest X-ray at the optimal instant of deepest inspiration. The degree of improvement is very different and depends on individual learning and reaction skills. Since the greatest improvements are already demonstrated after 5 minutes, the training program is particularly useful for radiology technicians with little experience taking chest X-rays in children in order to improve quality and diagnostic reliability. Further studies with evaluation of X-rays are necessary to prove the hypothesized improvement in the diagnostic reliability of chest X-rays—as a result of time optimization—caused by the training program.
